# Prevalence of Adult Asthma and History of Screening for Cancer Among US Adults: Results from 2016, 2018, 2020, and 2022 National Level Cross-Sectional Study

**DOI:** 10.3390/ijerph23010023

**Published:** 2025-12-23

**Authors:** Odele Rajpathy, Sanda Cristina Oancea

**Affiliations:** Department of Population Health, School of Medicine & Health Sciences, University of North Dakota, Room E 246, 1301 North Columbia Road, Grand Forks, ND 58202-9037, USA; odele.rajpathy@und.edu

**Keywords:** adult asthma, cancer screening, BRFSS, preventive health, chronic disease, prostate cancer, breast cancer, cervical cancer, colorectal cancer, health disparities

## Abstract

**Simple Summary:**

In the United States (US), cancer remains a leading cause of death, yet cancer screening tests can save lives by detecting it at an early stage. The present study aims to understand whether people with asthma and without a history of cancer are more likely to receive recommended cancer screenings compared to people without asthma. Both asthma and cancer are chronic conditions that contribute to long-term disease burden, which makes it crucial to examine how the presence of one may influence preventive care behaviors for the other. Using a 4-year national survey data, this study aimed to investigate the receipt of prostate, female breast, cervical, and colorectal cancer screening. Our study results show that US adults without a history of cancer and having asthma were actually more likely to follow cancer screening recommendations than those without asthma. In particular, men with asthma were more likely to receive prostate and colorectal screening, and women with asthma were more likely to receive breast, cervical, and colorectal screening, when compared to their counterparts without asthma. Education level and heavy drinking habits also influenced these patterns. These findings suggest that people with asthma may be more engaged in their healthcare including the receipt of cancer screening, and that tailored outreach may help further improve cancer screening across the US population.

**Highlights:**

**Public health relevance—How does this work relate to a public health issue?**

- This study addresses two prevalent chronic conditions—asthma and cancer—that together contribute substantially to morbidity and mortality in the United States, linking respiratory disease management with preventive oncology care.
- By examining national-level data across multiple cancer screening sites and survey years, the research highlights how individuals with adult asthma engage differently in preventive cancer screening behaviors, offering new insights into chronic disease interconnections at the population level.

**Public health significance—Why is this work of significance to public health?**

- The findings challenge prevailing assumptions that chronic illnesses such as asthma hinder preventive cancer screening uptake, showing instead that adults with asthma were significantly more likely to undergo cancer screening for prostate, breast, cervical, and colorectal cancers.
- This national analysis identifies education and alcohol consumption as key modifiers of screening behavior, underscoring the importance of behavioral and social determinants in shaping preventive health outcomes.

**Public health implications—What are the key implications or messages for practitioners, policy makers and/or researchers in public health?**

- This study suggests that chronic disease management visits—particularly for asthma—represent valuable opportunities for integrating cancer screening reminders and patient education within routine care.
- Policymakers and practitioners should leverage these findings to design integrated care models that link asthma follow-up with preventive service delivery, emphasizing health literacy and equitable access to screening for populations with chronic respiratory diseases.

**Abstract:**

Cancer is the second leading cause of death in the U.S., with 612,000 deaths estimated in 2023. Cancer screening (CS) reduces mortality through early detection, but the impact of chronic conditions like adult asthma (AA) on screening is less understood. This study explores the association between AA and uptake of prostate, breast, cervical, and colorectal CS using BRFSS 2016, 2018, 2020 and 2022 data. Weighted and adjusted multivariable logistic regression assessed the association between AA and CS across sex and age-based subgroups with significant effect modification testing and subsequent subgroup analyses. Results showed significantly higher CS adherence among individuals with AA across all four CS sites compared to counterparts without asthma (CCWA). Males (55–69 years old (YO)) with AA had 15% significantly higher weighted and adjusted odds (WAO) of prostate CS (95% CI: 1.04–1.27). Women (50–74 YO) with AA had 16% significantly higher WAO of breast CS (95% CI: 1.01–1.32), with non-depressed, heavy-drinking women showing 300% significantly higher WAO (95% CI: 2.20–7.22) of breast CS. Women (21–65 YO) with AA had 9% significantly higher WAO of cervical CS (95% CI: 1.02–1.17), with education significantly modifying the association (WAOR for college-educated women = 1.23, 95% CI: 1.11–1.36). When CCWA, colorectal CS showed significantly higher odds of 36% for men aged 50–75 (95% CI: 1.24–1.49) and 24% for women aged 50–75 (95% CI: 1.15–1.33). This is the first national study to examine the association between AA and uptake of prostate, female breast, cervical, and colorectal CS over four years. Individuals with AA had significantly greater odds of CS adherence than CCWA. Effect modification by heavy drinking and education suggests the need for targeted outreach and low-literacy interventions.

## 1. Introduction

Cancer, as of 2025, is one of the leading causes of illness and death worldwide [1]. In 2022, nearly 20 million new cancer cases were diagnosed, and approximately 9.7 million people died from cancer globally [2]. Lung, breast, colorectal, and prostate cancers account for the highest incidence and mortality worldwide [3]. The World Health Organization (WHO) projects this cancer burden to rise by 77% in cases and 90% in deaths by 2050 [4]. In the United States (U.S.) as of 2025, cancer remains the second most common cause of death, after cardiovascular disease [5]. The American Cancer Society (ACS) data reported an estimated 2 million new cancer cases and 612,000 cancer deaths in 2024 [6].

The most common risk factors for cancer include a combination of modifiable and non-modifiable risk factors such as age, heavy alcohol consumption, tobacco usage, unhealthy diet, chronic inflammation, obesity, sunlight, hormones, carcinogenic substances, immune-suppressive diseases, radiation, infectious agents and genetic factors (mutations in genes TP53, PIK3CA, PTEN, BRCA1 and BRCA2, etc.) [7].

Preventive cancer screening (CS) is hence a critical intervention for reducing both cancer incidence and mortality. In the U.S., the U.S. Preventive Services Task Force (USPSTF), a panel of national experts in the field of prevention and evidence-based medicine, issued evidence-based and age-specific CS recommendations for high-risk individuals during 2016–2022 such as mammography for breast CS in females ages 50–74 years, Pap/HPV testing for cervical CS in females ages 21–65 years, fecal occult blood testing and colonoscopy for colorectal CS in males and females ages 50–75 years, PSA test for prostate CS for males ages 55–69 years [8].

A nested case–control study of 1039 average-risk adults in four U.S. health plans during 2006–2008 found that screening colonoscopy was associated with a 71% reduction in risk of late-stage colorectal cancer, including a 64% reduction for right-sided colon cancer [9]. Women who underwent mammography screening experienced a 41% reduction in the risk of breast cancer mortality within 10 years of diagnosis, along with a 25% decrease in the incidence of advanced-stage breast cancers [10]. Cervical CS through regular HPV and Pap testing has been shown to reduce cervical cancer by 33% and 60%, respectively [11,12]. Regular PSA testing amongst individuals in a controlled trial showed a 20% reduction in prostate cancer mortality over follow-up periods [13].

CS holds an even greater importance among individuals with existing chronic diseases such as asthma, diabetes, cardiovascular disease, COPD, and obesity as they generally face higher morbidity and healthcare utilization when compared to individuals without chronic diseases. In a systematic analysis for a global burden of disease study in 2015, which investigated cause-specific mortality for 249 causes of death, chronic diseases have been reported to contribute to more than one-fifth of the risk for cancer incidence and more than one-third of the risk for cancer death [14]. More specifically prior epidemiological studies have consistently demonstrated an association between asthma and increased overall cancer risk over time, although the magnitude and site-specific patterns vary by population and disease severity. A longitudinal cohort data from the Melbourne Collaborative Cohort Study showed that a history of asthma was associated with a 25% increased risk of prostate cancer over a mean follow-up of 13.4 years from 1994 to 2007 [15]. In the Swedish Hospital Discharge Register, asthma patients hospitalized between 1965 and 2004 had a significantly elevated overall cancer risk with particularly high risks for gastrointestinal malignancies, including 70% significantly increased risk for stomach cancer and 99% increased risk for colon cancer [16]. More recently, contemporary U.S. real-world data from the OneFlorida+ Clinical Research Network during 2012–2020 demonstrated that adults with asthma had a 36% significantly higher risk of developing cancer compared with non-asthmatic controls with particularly elevated risks observed for lung, colorectal, liver, pancreatic, kidney, ovarian, bladder, hematologic malignancies, and melanoma [17].

In adults with adult asthma (AA), CS behavior presents a complex picture. Existing literature reports a 2.8% cancer detection rate among all cancer-screened participants but does not stratify results by asthma status [15]. While multiple articles report that there is an increase in lung CS in asthmatic adults due to frequent hospital visits for lung health monitoring [18,19,20,21], there is little evidence that these patients receive CS for non-respiratory cancers such as prostate, female breast, cervical, or colorectal. There also exists a literature gap as to whether AA-specific healthcare utilization translates into broader preventive behaviors in a large population followed over a long time-period.

The present study aims to examine the association between AA and the uptake of CS services in the U.S., focusing on female breast, cervical, prostate, and colorectal CS during the time interval 2016–2022. Specifically, this study seeks to address the following research questions:

1. At the national level, are U.S. adults with AA more likely to participate in recommended CS compared to adults without AA?
2. Are there any effect modifiers that influence the relationship between AA and CS uptake, and if yes, then how?

The results of this study will help to better understand whether chronic conditions like AA serve as barriers or motivators for preventive health behaviors such as CS. This insight can inform targeted public health strategies and policy interventions aimed at improving CS rates and reducing disparities among individuals with chronic respiratory illnesses.

## 2. Materials and Methods

### 2.1. Dataset

The Behavioral Risk Factor Surveillance System (BRFSS) is the largest, cross-sectional, national level survey conducted annually by the Center for Disease Control (CDC) since 1984. BRFSS collects annually health-related data from over 400,000 non-institutionalized U.S. adults aged 18 years and older using random-digit-dialed telephone interviews across all 50 states, the District of Columbia, and U.S. territories [22]. It is one of the most comprehensive health surveys available and is ideal for examining associations between chronic conditions, such as AA, and participation in preventive health services like CS. The BRFSS survey years selected for this study were 2016, 2018, 2020, and 2022. These even years were selected since AA and the CS under investigation were mandatory BRFSS core sections in these years, but not in the intervening odd years. Since prostate CS was no longer recommended after 2020 [23], it was included only as an optional module in 2022 and was therefore excluded from that year’s analyses.

### 2.2. Exposure of Interest: Adult Asthma

The exposure variable for this study was self-reported active AA. This was operationalized as a binary variable (Yes/No) and defined as the response to the BRFSS core question: “Do you still have asthma?” This question followed a prior screening item: “Has a doctor, nurse, or other health professional ever told you that you had asthma?”, but only the former was used to define current asthma status.

### 2.3. Outcomes of Interest: Female Breast, Prostate, Cervical, Female Colorectal, and Male Colorectal Cancer Screening

The outcome variables for this study were the receipt of site-specific CS female breast, cervical, prostate, female colorectal, and male colorectal cancers, also operationalized as binary variables (Yes/No) based on the corresponding sex and age recommendations given by the U.S. Preventive Services Task Force (USPSTF) for the years 2016–2022 [23,24]. A history of female breast CS was defined as a “Yes/No” based on the response to the core question, “Have you ever had a mammogram?”, among women aged 50–74 years [24]. Cervical CS was assessed using responses from women aged 21–65 years [24] who answered “Yes/No” to the question, “Ever had a PAP test?” or “Have you ever had an HPV test?”. A history of prostate CS was determined by a “Yes” response to the question, “Ever had a PSA test?”, among men aged 55–69 years [23]. Colorectal CS was defined for both men and women aged 50–75 years [24] based on a “Yes” response to the following questions: “Ever had blood stool test using home kit?” or “Ever had sigmoidoscopy/colonoscopy?”

### 2.4. Confounders of Interest

**Figure 1:**
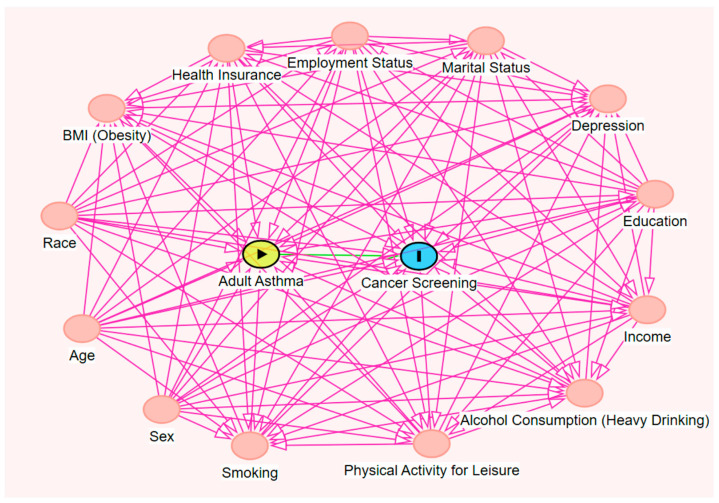
Directed Acyclic Graph (DAG) depicting the association between Adult Asthma and Cancer Screening.

The confounders included in this study were identified based on a review of the existing literature and were selected based on the confounder definition [25]. After identification, these variables were incorporated into a directed acyclic graph (DAG) [25] to determine the minimum sufficient set of confounders required for adjustment (Figure 1). The final set of confounders included: age [26,27], race/ethnicity [28,29], sex [30,31], regular cigarette smoking status [32,33], physical activity for leisure [34,35], heavy alcohol consumption [36,37], income [38,39], education [40,41], marital status [42,43], employment status [44,45], health insurance coverage [46,47], depression [48,49], and obesity [50,51] (Figure 1).

### 2.5. Final Study Sample

**Figure 2:**
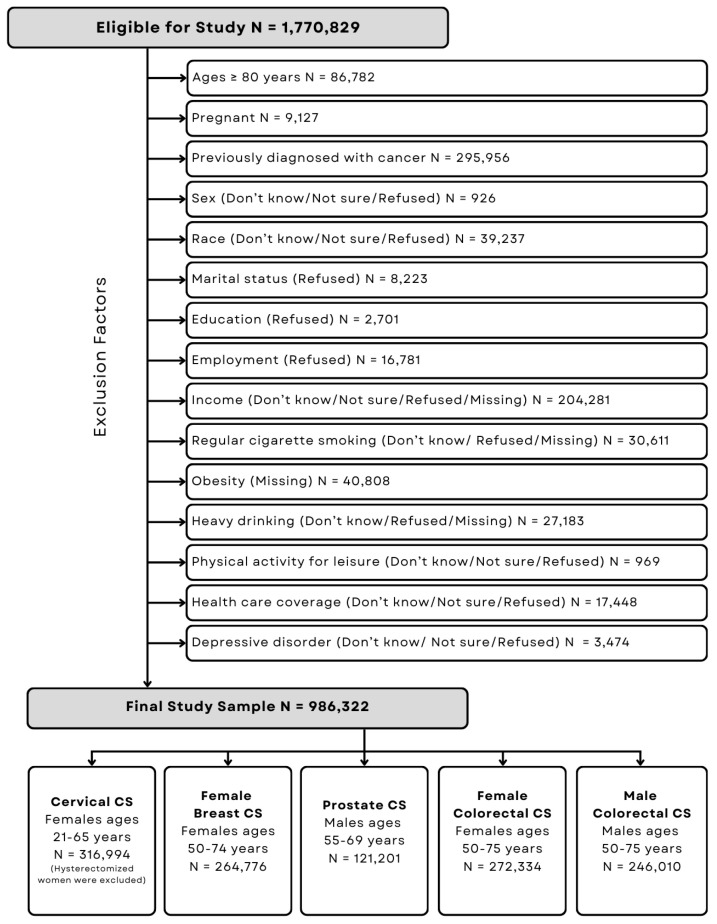
CONSORT diagram showing final study sample sizes after applying exclusion criteria.

For the four survey years under investigation (2016, 2018, 2020, 2022), a total of 1,770,829 BRFSS records were initially available for analysis. Several exclusion criteria were applied to derive the final study sample. Individuals aged 80 years and older were excluded due to the BRFSS’s post-2015 coding practice that imputes all such ages as 80, which limits age-based investigation past 80 years of age. Pregnant individuals were excluded to avoid potential bias in CS patterns due to pregnancy-related healthcare practices and physiological considerations [52]. Participants with a self-reported history of any cancer diagnosis were also excluded to minimize confounding, as these individuals may have heightened awareness of cancer risks and are more likely to undergo regular screenings as part of post cancer-diagnosis surveillance [53]. Among women ages 21–65 years, those who reported having had a hysterectomy were excluded, as they are no longer eligible for cervical CS per clinical guidelines. Furthermore, individuals with missing data or responses of “Don’t know/Not sure” or “Refused to Answer” for the variables included in the analyses were excluded. After all exclusions, the final analytic sample consisted of 986,322 individuals. This sample was further stratified by CS type and eligibility based on the United States Preventative Services Task Force (USPSTF) guideline-recommended age groups [23,24]. The breast CS subsample included 264,776 females aged 50–74 years [24]. The cervical CS sample comprised 316,994 females aged 21–65 years without a prior hysterectomy [24]. The prostate CS subsample included 121,201 males aged 55–69 years [23]. Colorectal CS eligibility included 272,334 females and 246,010 males, each between the ages of 50 and 75 years [24] (Figure 2).

### 2.6. Statistical Analyses

Descriptive statistics for categorical variables were summarized as unweighted frequencies and weighted percentages and 95% confidence interval (CI) for percentages, while continuous variables were summarized using weighted medians and interquartile ranges (IQR). For comparisons between groups, *p*-values for continuous variables (*p*-value *) were calculated using the Wald test, and *p*-values for categorical variables (*p*-value **) were calculated using the Rao-Scott chi-square test. Results for all variables, including demographic, socioeconomic, and health-related characteristics by prostate CS status, are presented in Supplemental Tables S1–S5.

Weighted unadjusted and adjusted logistic regression models were used to examine the association between active AA status and each CS outcome. Effect modification was assessed, and where statistically significant, subgroup analyses were conducted accordingly. All statistical analyses were performed using SAS version 9.4 (SAS Institute, Cary, NC, USA) and SAS OnDemand for Academics (SAS Institute, Cary, NC, USA). SAS-specific survey procedures (SURVEYMEANS, SURVEYFREQ, and SURVEYLOGISTIC) were used to account for the complex survey design. Statistical significance was defined as a two-sided *p*-value < 0.05.

## 3. Results

### 3.1. Descriptive Statistics

Among individuals eligible for CS across all sites, the majority were White, with a weighted prevalence ranging from 70.7% (95% CI: 70.33–71.06) among those eligible for cervical CS to 77.5% (95% CI: 76.88–78.15) among those eligible for prostate CS, and had health insurance, with a weighted prevalence range from 89.3% (95% CI: 89.02–89.53) among those eligible for cervical CS to 94.3% (95% CI: 94.06–94.52) among those eligible for breast CS. Depression was significantly higher in women, with the highest weighted prevalence of 24.7% (95% CI: 24.43–25.04) among women eligible for cervical CS, compared to the lowest weighted prevalence of 13.3% (95% CI: 12.96–13.53) in colorectal CS among men. Among women, being employed for wages or self-employed was most common among women eligible for cervical CS (weighted prevalence 67.9%, 95% CI: 67.57–68.28) and least common among women eligible for colorectal CS (weighted prevalence 47.6%, 95% CI: 47.13–48.02), in contrast to consistently high employment in men eligible for CS. The weighted prevalence of physical activity was consistently high, peaking at 78.9% (95% CI: 78.60–79.22) in cervical CS. The weighted prevalence for regular cigarette smoking was low, ranging from 14.7% (95% CI: 14.49–14.96) in cervical CS to 16.6% (95% CI: 16.26–16.92) in male colorectal CS, while the weighted prevalence of heavy alcohol consumption was lowest in female colorectal CS (6.3%, 95% CI: 6.07–6.48) and highest in cervical CS (8.0%, 95% CI: 7.79–8.17). The weighted prevalence of obesity ranged from 33.3% (95% CI: 32.90–33.59) in cervical CS to 36.6% (95% CI: 36.18–37.03) in male colorectal CS (Supplementary Tables S1–S5).

Among individuals who were eligible to be screened for cancer, the lowest AA prevalence was observed in males aged 55–69 (6.25%, 95% CI: 5.98–6.53), while the highest prevalence was among females aged 50–74 eligible for breast CS (12.37%, 95% CI: 12.08–12.67). Screening uptake was lowest among males eligible for prostate CS at a weighted prevalence of 53.3% (95% CI: 52.62–53.91) and highest among females eligible for breast CS at 94.6% (95% CI: 94.34–94.77). Comparing AA prevalence among individuals eligible for CS versus those who actually received CS revealed a consistent pattern of slightly higher AA prevalence among those screened across all cancer types. For prostate CS, AA prevalence increased from 6.25% (95% CI: 5.98–6.53) among eligible men to 6.31% (95% CI: 5.93–6.69) among those screened. Among women eligible for breast CS, AA prevalence rose from 12.37% (95% CI: 12.08–12.67) to 12.46% (95% CI: 12.15–12.76). Similarly, cervical CS participants had a slightly higher AA prevalence (11.66%, 95% CI: 11.42–11.91) compared to those eligible (11.55%, 95% CI: 11.32–11.78). In colorectal cancer, AA prevalence increased from 6.44% (95% CI: 6.23–6.64) to 6.87% (95% CI: 6.62–7.11) in males, and from 12.35% (95% CI: 12.06–12.64) to 12.87% (95% CI: 12.54–13.20) in females (Table 1).

### 3.2. Inferential Statistics

A total of 20 statistical models were run to investigate the association between AA and the investigated CS. The overall weighted and adjusted odds (WAO) of receiving CS among U.S. adults with AA were significantly greater compared with their counterparts without asthma (CCWA) across all four cancer sites examined namely prostate, female breast, cervical, and colorectal CS (Table 2).

**Prostate CS:** Among males aged 55–69 years, the weighted and adjusted odds ratio (WAOR) of prostate CS was 1.15 (95% CI: 1.04–1.27, *p* = 0.01), indicating 15% significantly greater weighted and adjusted odds (WAO) of undergoing prostate CS among individuals with AA when CCWA.

**Female Breast CS:** Among females aged 50–74 years, the WAOR for breast CS were 1.16 (95% CI: 1.01, 1.32; *p* = 0.04), indicating 16% significantly greater WAO of breast CS among individuals with AA when CCWA. Heavy drinking (*p* = 0.02) and depression (*p* = 0.09) were identified as significant and marginally significant effect modifiers of the association between AA and breast CS. As such, corresponding subgroup analyses were performed. Among adult females ages 50–74 years who are heavy drinkers, the WAO of breast CS were 107% significantly greater (95% CI: 1.32–3.25) in those with AA when CCWA. Adult females who were heavy drinkers and not depressed had 300% significantly greater WAO of undergoing breast CS (WAOR = 3.99; 95% CI: 2.20, 7.22; *p* < 0.0001) if they had AA when CCWA. Other heavy drinking-depression subgroups did not show significant associations between AA and breast CS.

**Cervical CS:** Among females aged 21–65 years, the WAOR for cervical CS was 1.09 (95% CI: 1.02, 1.17; *p* = 0.01), indicating 9% significantly greater WAO of cervical CS among individuals with AA when CCWA. Education was found to be a significant effect modifier (*p* = 0.002) of the association between AA and cervical CS. Adult females with AA who had completed some college or technical school had 14% significantly greater WAO (WAOR = 1.14; 95% CI: 1.01, 1.28; *p* = 0.04), and those with a college degree or more had 23% significantly greater WAO (WAOR = 1.23; 95% CI: 1.11, 1.36; *p* < 0.0001) of cervical CS when CCWA.

**Colorectal CS:** Among both males and females aged 50–75 years, AA was associated with significantly greater WAO of colorectal CS. Male adults with AA had WAOR of 1.36 (95% CI: 1.237, 1.487; *p* < 0.0001), and female adults had WAOR of 1.24 (95% CI: 1.15, 1.33; *p* < 0.0001), indicating 36% and 24% significantly greater odds of receiving colorectal CS, respectively, among individuals with AA when CCWA.

The BRFSS year was also assessed as a potential effect modifier for all four CS sites; however, no significant effect modification was observed across the study years.

## 4. Discussion

In this study, during the time interval 2016–2022, a significant positive association was found between having AA and receiving prostate, female breast, cervical and colorectal CS among U.S. adults. This association was strongest for colorectal cancer, where adults with asthma were 36% more likely to be screened among males and 24% more likely among females compared to those without AA. For breast cancer, women with AA had 16% higher odds of receiving CS, with a particularly large effect observed among heavy drinkers without depression. AA had relatively the weakest association with cervical CS, where women with AA were 9% more likely to be screened, especially among women with higher education levels. Men with AA were also more likely to undergo prostate CS, showing a 15% increase in CS compared to their CCWA. BRFSS survey year was not found to be a significant effect modifier in the association.

Historically, disparities in CS uptake have mostly been attributed to demographic and socioeconomic factors such as race, obesity, insurance status, family history, genetics, with specific chronic illness playing a lesser-discussed role [54,55]. The evidence that does exist shows mixed results regarding CS uptake in individuals with chronic diseases. A study conducted between 2008 and 2009 in four primary care clinics in two rural Oregon communities, among 3433 patients aged 55 and older with at least one clinic visit in the past two years, found that women with chronic lung disease and cardiovascular disease had significantly lower odds of being up-to-date with breast and cervical CS [56]. The present study specifically investigated the recommended CS by age interval for breast and cervical CS and evaluated the prevalence of CS at a national level. As such, the significant difference in sample size, national representativeness, and chronic disease under investigation may have led to differences in findings. Similarly, a 1991–1992 study of 4320 adults aged 52–64 in U.S. Midwestern practices found significantly lower odds of receiving mammograms, Pap smears, and colorectal screening among patients with diabetes, hypertension, heart disease, or lung disease [57]. Similar conclusions can be drawn here, in that the chronic diseases investigated by prior authors differ from asthma, and also the sample used is not a nationally representative sample. The CS recommendations during the 1990s were different from the more recent CS recommendations used in the present study. In a chart-review study conducted at an internal medicine clinic in a large teaching hospital during 1992 found that women over 50 with moderate to severe comorbidities were less likely to pursue receipt of mammography [58]. The former study was limited to one internal medicine clinic rather than a nationally representative sample, examined broad comorbidities rather than asthma specifically, and reflected screening practices from the early 1990s. In addition, their broad sample included women aged 50 and above, whereas the present study included women aged 50–74 defined by the 2016–2022 USPSTF breast CS guidelines. Another study among 1764 women older than 43 years who visited the general internal medicine or family practice clinics at the University of Alabama during 1995, found that each increase in comorbidity score was associated with a 17–20% decline in breast and cervical CS rates. These observed results were attributed to the tendency of both physicians and patients to prioritize management of existing illnesses over preventive care [59]. Marked differences can be noted here as well, such that the studied women were aged 50 and older in the United States during the 1990s. The study examined chronic conditions other than asthma, which limits comparability with our nationally representative, more recent sample. However, similarly to our study, a study using BRFSS data from 2020 and 2021, found that individuals with a history of chronic illnesses including respiratory diseases were significantly more likely to undergo colorectal CS [60]. Although limited to Oregon and West Virginia rather than the full national sample used in our analysis, the study was conducted in the same national surveillance system and within a similar time period as the present study thereby yielding similar results of significantly increased odds of colorectal CS in asthmatic individuals. However, some notable differences include that the exposures in the study encompassed chronic diseases, including asthma, as well as history of cancer, whereas the present study excluded individuals with a history of cancer to isolate the effect of asthma on CS. Additionally, the study included adults aged 45–75 years, while the present study focused on those aged 50–75 years as per the 2016–2022 USPSTF colorectal CS guidelines. Moreover, even fewer studies have directly examined asthma as a determinant of preventive cancer care at the U.S. national level.

Asthma is a well-recognized risk factor for lung cancer, with chronic airway inflammation and structural lung changes believed to contribute to increased carcinogenic potential over time [21,61]. Due to this strong association between asthma and lung cancer, most existing literature exploring the link between asthma and CS is focused primarily on lung CS [18,19,20]. Some studies suggest that asthma may also contribute to the development of cancers beyond the lungs, such as female breast, prostate, thyroid, and brain tumors, potentially due to the same systemic inflammation and immune dysregulation associated with a chronic respiratory disease [17,62]. Hence, it is important to consider screening for these cancers in individuals with asthma; however, there is currently a severe lack of literature specifically examining asthma’s specific role in influencing CS uptake for cancers beyond the lungs.

Interestingly, unlike some of the existing literature which focuses on asthma as a barrier to CS, the present study demonstrated that having asthma was associated with increased CS uptake across multiple CS types over a span of four years. This study findings may be influenced by several intersecting behavioral, clinical, and systemic factors. Asthma often requires regular medical management and follow-up due to common immunomodulatory medication regimens like corticosteroids, anticholinergics, and short-acting beta agonists [63], which increases patients’ contact with healthcare providers. It may be possible that this heightened interaction, including more frequent visits to pulmonologists and primary care physicians, may provide additional opportunities for healthcare providers to recommend preventive services such as CS, which may contribute to the higher rate of participation in national CS programs. Several studies report increased healthcare utilization among individuals diagnosed with asthma compared to those without asthma. Healthcare utilization is a known facilitator of higher CS uptake, including primary care visits, emergency department usage, and hospitalizations. A retrospective observational study of over 149,000 patients from Baylor Scott & White Health, a large integrated healthcare system in North/Central Texas during 2016–2018 showed high rates of emergency department and hospital use, specifically 52.9% and 26.7%, respectively, among asthma patients [64]. Similarly, a community-based study of 1678 adults in Hampton Roads found that individuals with asthma were significantly more likely to frequently utilize primary care, emergency departments, and hospitalizations compared to non-asthma participants [65]. Additionally, a U.S. claims database analysis during 2010–2011 involving over 220,000 asthma patients indicate that exacerbation frequency, ED visits, and re-admissions were substantially higher in those with more severe asthma statuses [66]. While our study could not directly assess asthma severity or detailed healthcare utilization metrics, these findings suggest that both the severity of asthma and higher frequency of healthcare encounters may contribute to increased CS uptake.

Both asthma and cancer are health conditions characterized by disruptions in immune function. Asthma involves chronic inflammation driven by an overactive immune response to environmental triggers [67], while cancer often arises due to failures in immune function and cell growth regulation, allowing abnormal cells to evade detection and proliferate [68]. In turn, people with asthma may often be more attuned to health messaging, especially when related to inflammation or immune function, which may promote adherence to screening recommendations [69].

Analysis of effect modifiers also provided important context for understanding the mechanisms driving the observed associations between asthma prevalence and CS uptake. For breast cancer, the strong positive association among heavy drinkers without depression could reflect higher medical surveillance in this subgroup. Heavy alcohol use is a well-established risk factor for breast cancer, which often prompts clinicians to emphasize screening adherence during routine care visits [70]. However, this effect was not seen among heavy drinkers with depression, which could mean that simultaneously having comorbid mental health conditions may counteract increased medical surveillance by reducing patient motivation, perceived self-efficacy, or engagement with preventive care, as documented in prior research linking depression to lower CS rates [71,72,73]. For cervical cancer, women possessing some college or higher education showed significantly greater odds of screening uptake, and this is a reiteration of how higher education levels are frequently associated with improved health literacy, stronger patient–provider communication, and greater capacity to navigate preventive health systems [74]. In contrast, prostate and colorectal CS showed consistently significantly higher odds of CS among adults with asthma CCWA regardless of stratification, which suggests that the link between asthma and increased CS in these contexts may be driven less by socioeconomic or behavioral differences and more by systemic factors, such as the frequency of medical encounters.

### 4.1. Study Limitations

The limitations of this study include its cross-sectional design, which restricts the ability to draw causal inferences. Asthma status was self-reported based on physician-obtained diagnosis. Although this method is widely used in population surveillance, it may lead to misclassification because this study was not able to independently verify individual diagnoses via spirometry or clinical records. Similarly, the BRFSS self-reported data on CS may introduce recall bias or additional misclassification. This study was also unable to differentiate between AA severity levels, which could influence healthcare utilization patterns and attitudes toward preventive care such as CS. The BRFSS also does not capture data on the actual healthcare provider-level recommendations, which may also influence CS uptake.

### 4.2. Study Strengths

However, this study has several strengths that should be noted, such as the use of a nationally representative study sample, a very large sample size, as well as the inclusion of multiple CS types across different organ systems. This study also used four years of BRFSS data, further increasing generalizability and which is not commonly seen in other similar studies. The analysis adjusted for a wide range of DAG selected confounders, which increased the robustness of the observed associations and helped close potentially biasing pathways.

### 4.3. Directions for Future Research

Future studies should investigate longitudinal patterns of CS adherence among individuals with AA, particularly using observational cohort designs to explore causal pathways. It is also important to evaluate whether AA severity, medication adherence, or coexisting comorbidities influence CS behavior. Qualitative studies could also offer valuable insights into patient and provider perspectives on preventive care prioritization in the context of managing chronic respiratory diseases. Further research should also explore geographic disparities in this association, particularly in underserved populations.

### 4.4. Implications for Public Health

These study results suggest that individuals with AA may represent a unique population with increased engagement in preventive health behaviors, potentially due to their routine healthcare interactions. This emphasizes the importance of leveraging chronic disease management platforms as access points for preventive care interventions, including CS. Recognizing AA as a facilitator rather than a barrier to screening, challenges conventional assumptions in chronic illness care and opens opportunities to design integrated care models that align disease management with preventive service delivery. Health systems and policy-makers should consider targeted CS reminders or educational interventions during routine asthma management encounters. Reflecting on these results, which report significantly higher odds of breast CS among asthmatic heavy drinkers without depression, this indicates that this subgroup, who are often viewed as high-risk for disengagement may actually be more receptive to preventive services when engaged through chronic disease care. This may actually be an opportunity for integrating CS prompts within routine asthma care visits specifically tailored to individuals with high-risk health behaviors such as heavy alcohol use. Similarly, there is also a critical gap among individuals with lower education levels, who may not be benefiting equally from screening recommendations despite similar clinical opportunities. To address this disparity, public health interventions must prioritize health literacy, community-based education, and access-enhancing strategies for CS, particularly among women with asthma and limited formal education.

## 5. Conclusions

Contrary to prevailing assumptions and popular belief that chronic illnesses such as adult asthma hinder preventive care uptake [75], this study demonstrates that adults with AA were significantly more likely to undergo CS across several CS types, particularly prostate, cervical, female breast, and colorectal cancers. This positive association may be driven by higher healthcare engagement, increased patient awareness, or provider recommendations. These findings bring to light the need for further research and integrated healthcare strategies that capitalize on respiratory chronic disease follow-ups to improve CS adherence at the population level.

## Figures and Tables

**Table 1:** Weighted Prevalence of Asthma and Cancer Screening Uptake by Sex, Screening Site, and Age Group in U.S. Adults.

Sex	Cancer Screening (CS) Site	Age Range (Years)	Final Sample (Unweighted N)	Have Asthma (Unweighted N)	% Who Have Asthma Weighted % (95% CI)	Received CS (Unweighted N)	Received CS Weighted % (95% CI)	% of Those Who Received CS Who Have Asthma Weighted % (95% CI)
Male	Prostate	55–69	121,201	7934	6.25 (5.98–6.53)	66,507	53.27 (52.62–53.91)	6.31 (5.93–6.69)
Female	Breast	50–74	264,776	32,793	12.37 (12.08–12.67)	251,283	94.56 (94.34–94.77)	12.46 (12.15–12.76)
Female	Cervical	21–65	316,994	37,716	11.55 (11.32–11.78)	277,790	84.42 (84.13–84.70)	11.66 (11.42–11.91)
Male	Colorectal	50–75	246,010	16,343	6.44 (6.23–6.64)	182,834	70.66 (70.23- 71.08)	6.87 (6.62–7.11)
Female	Colorectal	50–75	272,334	33,593	12.35 (12.06–12.64)	212,729	75.17 (74.77–75.57)	12.87 (12.54–13.20)

Abbreviations: CI = Confidence Interval; N = Sample size.

**Table 2:** Weighted Unadjusted and Adjusted Odds Ratios for Cancer Screening by Site, Sex, Age, and Effect Modifiers in U. S. Adults with Asthma (AA) Compared with their Counterparts without Asthma (CCWA).

Cancer Screening Site	Sex	Age Range	Effect Modifier	WUOR	95% CI for WUOR	WUOR *p*-Value	WAOR	95% CI for WAOR	WAOR *p*-Value
Prostate Cancer	Male	55–69	Overall	1.02	0.93–1.12	0.66	1.15	1.04–1.27	0.01 *
Breast Cancer	Female	50–74	Overall	1.16	1.02–1.31	0.03 *	1.16	1.01–1.32	0.04 *
			*Non-heavy Drinkers*				1.12	0.97–1.28	0.12
			*Heavy drinkers*				2.07	1.32–3.25	0.001 *
			*Heavy drinkers who are depressed*				0.92	0.48–1.77	0.8
			*Heavy drinkers who are not depressed*				3.99	2.20–7.22	<0.0001 *
			*Non-heavy drinkers who are depressed*				1.04	0.84–1.28	0.72
			*Non-heavy drinkers who are not depressed*				1.17	0.98–1.40	0.08
Cervical Cancer	Female	21–65	Overall	1.07	1.01–1.15	0.03 *	1.09	1.02–1.17	0.01 *
			*Less than high school graduate*				1.12	0.91–1.38	0.27
			*High school graduate or GED*				0.89	0.77–1.02	0.08
			*Some college or technical school*				1.14	1.01–1.28	0.04 *
			*College graduate or more*				1.23	1.11–1.36	<0.0001 *
Colorectal Cancer	Male	50–75	Overall	1.29	1.19–1.40	<0.0001 *	1.36	1.24–1.49	<0.0001 *
Colorectal Cancer	Female	50–75	Overall	1.23	1.15–1.31	<0.0001 *	1.24	1.15–1.33	<0.0001 *

Abbreviations: WUOR = Weighted and unadjusted odds ratios; WAOR = Weighted and adjusted odds ratios; CI = Confidence Intervals. Footnote: * Values are significant at the 0.05 level.

## Data Availability

All BRFSS data are publicly available.

## Supplementary Materials

The following supporting information can be downloaded at: https://www.mdpi.com/article/10.3390/ijerph23010023/s1: Table S1: Weighted Distribution of Sample Characteristics by Prostate Cancer Screening Status Among U.S. Males Aged 55–69; Table S2: Weighted Distribution of Sample Characteristics by Breast Cancer Screening Status Among U.S. Females Aged 50–74; Table S3: Weighted Distribution of Sample Characteristics by Cervical Cancer Screening Status Among U.S. Females Aged 21–65; Table S4: Weighted Distribution of Sample Characteristics by Colorectal Cancer Screening Status Among U.S. Males Aged 50–75; Table S5: Weighted Distribution of Sample Characteristics by Colorectal Cancer Screening Status Among U.S. Females Aged 50–75.

## Abbreviations

The following abbreviations are used in this manuscript:

AA	Adult Asthma
CS	Cancer Screening
US	United States
BRFSS	Behavioral Risk Factor Surveillance System
WAUR	Weighted and Unadjusted Odds Ratio
WAOR	Weighted and Adjusted Odds Ratio
CI	Confidence Intervals
CCWA	Compared to Counterparts Without Asthma
