# Supplementary material for: Prevalence of Adult Asthma and History of Screening for Cancer Among US Adults: Results from 2016, 2018, 2020, and 2022 National Level Cross-Sectional Study
Source: Int J Environ Res Public Health. 2025 Dec 23;23(1):23. doi: 10.3390/ijerph23010023 (PMC12840605; doi:10.3390/ijerph23010023)
Supplement: Supplementary file 1 [file ijerph-23-00023-s001.zip › Table S1.pdf]

Table S1: Weighted Distribution of Sample Characteristics by Prostate Cancer Screening Status Among U.S. Males Aged 55–69

	Overall Counts (N = 121,201)			Screened for Prostate Cancer: Yes (N = 66,507)		Screened for Prostate Cancer: No (N = 54,694)		
	Unweighted Counts	Weighted Median	Weighted IQR	Weighted Median	Weighted IQR	Weighted Median	Weighted IQR	P Value*
**Age at survey**	121,201	60.7	57.46 - 64.18	61.37	58.09 - 64.74	59.87	56.90 - 63.50	<.0001

	Overall Counts (N = 121,201)			Screened for Prostate Cancer: Yes (N = 66,507)		Screened for Prostate Cancer: No (N = 54,694)		
	Unweighted Counts	Weighted Percent	95% CI for Percent	Weighted Percent	95% CI for Percent	Weighted Percent	95% CI for Percent	P Value**
**Currently Have Asthma**								
Yes	7,934	6.25	5.98 - 6.53	6.31	5.93 - 6.69	6.19	5.79 - 6.59	0.66
No	113,267	93.75	93.47 - 94.02	93.69	93.31 - 94.07	93.81	93.41 - 94.21	
**Race**								
White, Non-Hispanic	101,894	77.51	76.88 - 78.15	78.97	78.12 - 79.82	75.85	74.87 - 76.83	<.0001
Black, Non-Hispanic	9,174	11.75	11.30 - 12.21	12.05	11.42 - 12.69	11.42	10.77 - 12.06	
Other Races	10,133	10.73	10.19 - 11.28	8.98	8.29 - 9.66	12.73	11.86 - 13.60	
**Education**								
Less than high school graduate	7,679	12.38	11.84 - 12.92	8.07	7.49 - 8.66	17.29	16.37 - 18.21	<.0001
High school graduate or GED	34,574	28.47	27.92 - 29.02	25.35	24.62 - 26.08	32.02	31.18 - 32.87	
Some college or technical school	32,506	30.35	29.73 - 30.98	30.96	30.14 - 31.79	29.66	28.71 - 30.61	
College graduate or more	46,442	28.8	28.28 - 29.32	35.62	34.85 - 36.38	21.03	20.37 - 21.69	
**Employment Status**								
Employed for wages or self-employed	67,667	56.2	55.56 - 56.84	56.17	55.32 - 57.01	56.24	55.25 - 57.23	<.0001
Homemaker, student, or retired	36,584	27.99	27.41 - 28.57	31.59	30.82 - 32.36	23.9	23.01 - 24.79	
Out of work	5,247	5.04	4.74 - 5.34	3.84	3.46 - 4.23	6.41	5.94 - 6.88	
Unable to work	11,703	10.76	10.34 - 11.18	8.41	7.90 - 8.92	13.45	12.77 - 14.14	
**Income**								
<$15,000	10,723	9.76	9.31 - 10.20	6.32	5.88 - 6.76	13.67	12.88 - 14.47	<.0001
$15,000 to less than $25,00	16,035	13.57	13.13 - 14.02	10.55	10.02 - 11.08	17.01	16.28 - 17.74	
$25,000 to less than $35,00	10,665	8.85	8.43 - 9.27	7.28	6.82 - 7.74	10.65	9.92 - 11.37	
$35,000 to less than $50,00	15,969	12.44	12.04 - 12.85	11.91	11.38 - 12.44	13.05	12.42 - 13.68	
$50,000 or more	67,809	55.38	54.73 - 56.02	63.94	63.12 - 64.76	45.62	44.66 - 46.58	
**Marital Status**								
Married or member of an unmarried couple	79,258	68.6	68.02 - 69.19	74.37	73.63 - 75.11	62.03	61.10 - 62.95	<.0001
Never married	12,571	9.07	8.72 - 9.43	7.08	6.63 - 7.53	11.34	10.78 - 11.91	
Separated, divorced, or widowed	29,372	22.33	21.81 - 22.85	18.55	17.90 - 19.20	26.63	25.80 - 27.46	
**Health Insurance Coverage**								
Yes	113,478	92.27	91.85 - 92.69	95.51	95.10 - 95.93	88.57	87.82 - 89.32	<.0001
No	7,723	7.73	7.31 - 8.15	4.49	4.07 - 4.90	11.43	10.68 - 12.18	
**Smoking Status**								
Current smoker	20,296	17.7	17.21 - 18.20	12.73	12.16 - 13.31	23.37	22.54 - 24.20	<.0001
Former smoker	41,872	34.51	33.89 - 35.12	36.09	35.28 - 36.89	32.7	31.77 - 33.63	
Never smoker	59,033	47.79	47.14 - 48.44	51.18	50.33 - 52.03	43.92	42.94 - 44.90	
**Physical Activity for Leisure in Past 30 Days**								
Yes	92,604	75.26	74.69 - 75.82	79.3	78.59 - 80.00	70.65	69.76 - 71.55	<.0001
No	28,597	24.74	24.18 - 25.31	20.7	20.00 - 21.41	29.35	28.45 - 30.24	
**Heavy Alcohol Consumption (Male > 14 drinks/week; Female > 7 drinks/week)**								
Yes	8,230	6.65	5.34 - 6.95	5.76	5.40 - 6.13	7.65	7.16 - 8.15	<.0001
No	112,971	93.35	93.05 - 93.66	94.24	93.87 - 94.60	92.35	91.85 - 92.84	
**Depression**								
Yes	16,988	13.64	13.21 - 14.07	13.6402	13.06 - 14.22	13.64	12.99 - 14.29	1
No	104,213	86.36	85.93 - 86.79	86.36	85.78 - 86.94	86.36	85.71 - 87.01	
**Obesity**								
Obese	43,683	35.85	35.23 - 36.47	36.35	35.53 - 37.18	35.28	34.33 - 36.22	0.09
Not obese	77,518	64.15	63.53 - 64.77	63.65	62.82 - 64.47	64.72	63.78 - 65.67	

Footnotes:

* P-value calculated using the Wald test.

** P-value calculated using the Rao–Scott chi-square test.
