# Supplementary material for: Prevalence of Adult Asthma and History of Screening for Cancer Among US Adults: Results from 2016, 2018, 2020, and 2022 National Level Cross-Sectional Study
Source: Int J Environ Res Public Health. 2025 Dec 23;23(1):23. doi: 10.3390/ijerph23010023 (PMC12840605; doi:10.3390/ijerph23010023)
Supplement: Supplementary file 1 [file ijerph-23-00023-s001.zip › Table S2.pdf]

Table S2: Weighted Distribution of Sample Characteristics by Breast Cancer Screening Status Among U.S. Females Aged 50-74

	Overall Counts (N = 264,776)			Screened for Breast Cancer Yes (N = 251,283)		Screened for Breast Cancer No (N = 13,493)		
	**Unweighted Counts**	**Weighted Median**	**Weighted IQR**	**Weighted Median**	**Weighted IQR**	**Weighted Median**	**Weighted IQR**	**P Value***
**Age at survey**	264,776	59.58	54.06 - 65.37	59.71	54.19 - 65.51	56.94	52.35 - 62.68	<.0001

	Overall Counts (N = 264,776)			Screened for Breast Cancer Yes (N = 251,283)		Screened for Breast Cancer No (N = 13,493)		P Value**
	**Unweighted Counts**	**Weighted Percent**	**95% CI for Percent**	**Weighted Percent**	**95% CI for Percent**	**Weighted Percent**	**95% CI for Percent**	
**Currently Have Asthma**								
Yes	32,793	12.37	12.08 - 12.67	12.46	12.15 - 12.76	10.96	9.75 - 12.17	0.02
No	231,983	87.63	87.33 - 87.92	87.54	87.24 - 87.85	89.04	87.83 - 90.25	
**Race**								
White, Non-Hispanic	217,469	76.17	75.73 - 76.62	76.33	75.88 - 76.78	73.53	71.35 - 75.70	<.0001
Black, Non-Hispanic	26,935	13.64	13.33 - 13.96	13.72	13.40 - 14.05	12.32	10.99 - 13.65	
Other Races	20,372	10.18	9.80 - 10.56	9.95	9.57 - 10.34	14.15	12.09 - 16.22	
**Education**								
Less than high school graduate	13,873	10.22	9.88 - 10.57	9.88	9.53 - 10.22	16.28	14.42 - 18.14	<.0001
High school graduate or GED	66,853	26.31	25.93 - 26.70	25.96	25.57 - 26.36	32.4	30.59 - 34.21	
Some college or technical school	77,786	33.05	32.62 - 33.48	33.21	32.77 - 33.65	30.29	28.43 - 32.15	
College graduate or more	106,264	30.41	30.03 - 30.79	30.95	30.56 - 31.34	21.03	19.26 - 22.79	
**Employment Status**								
Employed for wages or self-employed	122,282	48.43	47.98 - 48.88	48.31	47.85 - 48.77	50.52	48.44 - 52.60	<.0001
Homemaker, student, or retired	106,205	36.04	35.61 - 36.47	36.42	35.98 - 36.86	29.47	27.64 - 31.30	
Out of work	9,749	4.56	4.35 - 4.77	4.43	4.22 - 4.65	6.78	5.77 - 7.80	
Unable to work	26,540	10.97	10.70 - 11.24	10.84	10.57 - 11.11	13.23	11.96 - 14.49	
**Income**								
<$15,000	26,166	10.5	10.21 - 10.78	10.12	9.83 - 10.40	17.07	15.35 - 18.79	<.0001
$15,000 to less than $25,00	39,698	14.79	14.47 - 15.10	14.42	14.10 - 14.74	21.09	19.45 - 22.75	
$25,000 to less than $35,00	28,035	10.27	10.01 - 10.54	10.17	9.90 - 10.44	12.09	10.86 - 13.31	
$35,000 to less than $50,00	36,848	12.72	12.42 - 13.02	12.69	12.39 - 13.00	13.17	11.77 - 14.57	
$50,000 or more	134,029	51.73	51.28 - 52.17	52.6	52.14 - 53.06	36.58	34.57 - 38.59	
**Marital Status**								
Married or member of an unmarried couple	150,203	59.9	59.46 - 60.34	60.27	59.83 - 60.72	53.45	51.37 - 55.52	<.0001
Never married	22,016	8.03	7.78 - 8.29	7.8	7.55 - 8.06	12	10.46 - 13.55	
Separated, divorced, or widowed	92,557	32.06	31.65 - 32.48	31.92	31.50 - 32.35	34.55	32.64 - 36.45	
**Health Insurance Coverage**								
Yes	253,061	94.29	94.06 - 94.52	94.98	94.76 - 95.19	82.41	80.62 - 84.20	<.0001
No	11,715	5.71	5.48 - 5.94	5.02	4.81 - 5.24	17.59	15.80 - 19.38	
**Smoking Status**								
Current smoker	38,792	14.94	14.64 - 15.24	14.24	13.94 - 14.54	27.06	25.218 - 28.90	<.0001
Former smoker	72,931	26.47	26.08 - 26.85	26.75	26.36 - 27.15	21.51	20.05 - 22.97	
Never smoker	153,053	58.59	58.16 - 59.03	59.01	58.56 - 59.45	51.43	49.36 - 53.51	
**Physical Activity for Leisure in Past 30 Days**								
Yes	194,958	72.36	71.96 - 72.77	72.78	72.36 - 73.19	65.16	63.09 - 67.23	<.0001
No	69,818	27.64	27.23 - 28.04	27.22	26.81 - 27.64	34.84	32.77 - 36.91	
**Heavy Alcohol Consumption (Male > 14 drinks/week; Female > 7 drinks/week)**								
Yes	17,201	6.33	6.12 - 6.54	6.28	6.07 - 6.50	7.23	6.27 - 8.19	<.0001
No	247,575	93.67	93.46 - 93.88	93.72	93.50 - 93.93	92.77	91.81 - 93.73	
**Depression**								
Yes	64,629	23.83	23.47 - 24.20	24.06	23.68 - 24.43	19.94	18.50 - 21.38	<.0001
No	200,147	76.17	75.80 - 76.53	75.94	75.57 - 76.32	80.06	78.62 - 81.50	
**Obesity**								
Obese	94,986	36.12	35.69 - 36.55	36.35	35.91 - 36.79	32.03	30.18 - 33.89	<.0001
Not obese	169,790	63.88	63.46 - 64.31	63.65	63.21 - 64.09	67.97	66.11 - 69.82	

Footnotes:
* P-value calculated using the Wald test.
** P-value calculated using the Rao–Scott chi-square test.
