# Supplementary material for: Prevalence of Adult Asthma and History of Screening for Cancer Among US Adults: Results from 2016, 2018, 2020, and 2022 National Level Cross-Sectional Study
Source: Int J Environ Res Public Health. 2025 Dec 23;23(1):23. doi: 10.3390/ijerph23010023 (PMC12840605; doi:10.3390/ijerph23010023)
Supplement: Supplementary file 1 [file ijerph-23-00023-s001.zip › Table S3.pdf]

**Table S3:** Weighted Distribution of Sample Characteristics by Cervical Cancer Screening Status Among U.S. Females Aged 21–65

| | Overall Counts ( N = 316,994 ) | | | Screened for Cervical Cancer: Yes (N = 277,790 ) | | Screened for Cervical Cancer: No (N = 39,204 ) | | |
|---|---|---|---|---|---|---|---|---|
| | Unweighted Counts | Weighted Median | Weighted IQR | Weighted Median | Weighted IQR | Weighted Median | Weighted IQR | P Value* |
| **Age at survey** | 316,994 | 39.32 | 29.93 - 51.01 | 40.44 | 31.19 - 51.75 | 32.46 | 23.95 - 44.33 | <.0001 |

| | Overall Counts ( N = 316,994 ) | | | Screened for Cervical Cancer: Yes (N = 277,790 ) | | Screened for Cervical Cancer: No (N = 39,204 ) | | |
|---|---|---|---|---|---|---|---|---|
| | Unweighted Counts | Weighted Percent | 95% CI for Percent | Weighted Percent | 95% CI for Percent | Weighted Percent | 95% CI for Percent | P Value** |
| **Currently Have Asthma** | | | | | | | | |
| *Yes* | 37,716 | 11.55 | 11.32 - 11.78 | 11.66 | 11.42 - 11.91 | 10.95 | 10.35 - 11.54 | <.0001 |
| *No* | 279,278 | 88.45 | 88.22 - 88.68 | 88.34 | 88.09 - 88.58 | 89.05 | 88.46 - 89.65 | |
| **Race** | | | | | | | | |
| *White, Non-Hispanic* | 247,051 | 70.69 | 70.33 - 71.06 | 72.56 | 72.17 - 72.94 | 60.59 | 59.53 - 61.66 | <.0001 |
| *Black, Non-Hispanic* | 33,663 | 14.66 | 14.39 - 14.92 | 14.32 | 14.03 - 14.60 | 16.5 | 15.74 - 17.26 | |
| *Other Races* | 36,280 | 14.65 | 14.33 - 14.97 | 13.13 | 12.80 - 13.46 | 22.91 | 21.91 - 23.91 | |
| **Education** | | | | | | | | |
| *Less than high school graduate* | 14,452 | 8.8 | 8.53 - 9.07 | 8.42 | 8.14 - 8.70 | 10.86 | 10.05 - 11.67 | <.0001 |
| *High school graduate or GED* | 64,616 | 21.54 | 21.23 - 21.85 | 20.72 | 20.40 - 21.05 | 26 | 25.08 - 26.92 | |
| *Some college or technical school* | 87,911 | 32.2 | 31.84 - 32.55 | 32.12 | 31.74 - 32.50 | 32.58 | 31.57 - 33.59 | |
| *College graduate or more* | 150,015 | 37.46 | 37.13 - 37.79 | 38.73 | 38.37 - 39.10 | 30.56 | 29.69 - 31.42 | |
| **Employment Status** | | | | | | | | |
| *Employed for wages or self-employed* | 220,286 | 67.93 | 67.57 - 68.28 | 68.63 | 68.26 - 69.01 | 64.12 | 63.11 - 65.14 | <.0001 |
| *Homemaker, student, or retired* | 57,732 | 19.64 | 19.33 - 19.95 | 19.19 | 18.86 - 19.52 | 22.06 | 21.13 - 22.99 | |
| *Out of work* | 17,729 | 6.56 | 6.37 - 6.76 | 6.35 | 6.14 - 6.56 | 7.73 | 7.22 - 8.24 | |
| *Unable to work* | 21,247 | 5.87 | 5.71 - 6.03 | 5.83 | 5.65 - 6.00 | 6.09 | 5.65 - 6.54 | |
| **Income** | | | | | | | | |
| *<$15,000* | 27,715 | 9.56 | 9.32 - 9.80 | 9.06 | 8.82 - 9.30 | 12.28 | 11.46 - 13.09 | <.0001 |
| *$15,000 to less than $25,00* | 41,976 | 14.34 | 14.07 - 14.60 | 13.89 | 13.61 - 14.17 | 16.76 | 15.99 - 17.54 | |
| *$25,000 to less than $35,00* | 30,023 | 9.8 | 9.59 - 10.01 | 9.09 | 8.87 - 9.32 | 13.63 | 12.97 - 14.29 | |
| *$35,000 to less than $50,00* | 39,298 | 12.12 | 11.88 - 12.36 | 11.92 | 11.67 - 12.17 | 13.22 | 12.53 - 13.91 | |
| *$50,000 or more* | 177,982 | 54.18 | 53.81 - 54.55 | 56.04 | 55.65 - 56.43 | 44.11 | 43.08 - 45.14 | |
| **Marital Status** | | | | | | | | |
| *Married or member of an unmarried couple* | 188,458 | 58.17 | 57.80 - 58.53 | 60.58 | 60.20 - 60.97 | 45.09 | 44.04 - 46.14 | <.0001 |
| *Never married* | 64,902 | 25.18 | 24.85 - 25.50 | 22.21 | 21.87 - 22.55 | 41.25 | 40.23 - 42.28 | |
| *Separated, divorced, or widowed* | 63,634 | 16.66 | 16.39 - 16.92 | 17.21 | 16.93 - 17.49 | 13.66 | 12.97 - 14.35 | |
| **Health Insurance Coverage** | | | | | | | | |
| *Yes* | 290,913 | 89.27 | 89.02 - 89.53 | 89.86 | 89.59 - 90.13 | 86.08 | 85.31 - 86.85 | <.0001 |
| *No* | 26,081 | 10.73 | 10.47 - 10.98 | 10.14 | 9.87 - 10.41 | 13.92 | 13.15 - 14.69 | |
| **Smoking Status** | | | | | | | | |
| *Current smoker* | 49,577 | 14.72 | 14.49 - 14.96 | 15.17 | 14.92 - 15.43 | 12.28 | 11.67 - 12.89 | <.0001 |
| *Former smoker* | 63,763 | 17.82 | 17.55 - 18.09 | 18.77 | 18.47 - 19.06 | 12.68 | 12.05 - 13.31 | |
| *Never smoker* | 203,654 | 67.46 | 67.13 - 67.79 | 66.06 | 65.70 - 66.42 | 75.04 | 74.21 - 75.87 | |
| **Physical Activity for Leisure in Past 30 Days** | | | | | | | | |
| *Yes* | 252,152 | 78.91 | 78.60 - 79.22 | 79.58 | 79.26 - 79.91 | 75.24 | 74.34 - 76.15 | <.0001 |
| *No* | 64,842 | 21.09 | 20.78 - 21.40 | 20.42 | 20.09 - 20.74 | 24.76 | 23.85 - 25.66 | |
| **Heavy Alcohol Consumption (Male > 14 drinks/week; Female > 7 drinks/week)** | | | | | | | | |
| *Yes* | 25,716 | 7.98 | 7.79 - 8.17 | 8.05 | 7.85 - 8.25 | 7.59 | 7.10 - 8.09 | 0.1 |
| *No* | 291,278 | 92.02 | 91.83 - 92.21 | 91.95 | 91.75 - 92.15 | 92.41 | 91.91 - 92.90 | |
| **Depression** | | | | | | | | |
| *Yes* | 81,463 | 24.73 | 24.43 - 25.04 | 25 | 24.68 - 25.33 | 23.27 | 22.47 - 24.06 | 0.0001 |
| *No* | 235,531 | 75.27 | 74.96 - 75.57 | 75 | 74.67 - 75.32 | 76.73 | 75.94 - 77.53 | |
| **Obesity** | | | | | | | | |
| *Obese* | 107,055 | 33.25 | 32.90 - 33.59 | 33.26 | 32.89 - 33.63 | 33.16 | 32.17 - 34.15 | 0.8472 |
| *Not obese* | 209,939 | 66.75 | 66.41 - 67.10 | 66.74 | 66.37 - 67.11 | 66.84 | 65.85 - 67.83 | |

**Footnotes:**

* P-value calculated using the Wald test.

** P-value calculated using the Rao–Scott chi-square test.
