# Supplementary material for: Prevalence of Adult Asthma and History of Screening for Cancer Among US Adults: Results from 2016, 2018, 2020, and 2022 National Level Cross-Sectional Study
Source: Int J Environ Res Public Health. 2025 Dec 23;23(1):23. doi: 10.3390/ijerph23010023 (PMC12840605; doi:10.3390/ijerph23010023)
Supplement: Supplementary file 1 [file ijerph-23-00023-s001.zip › Table S4.pdf]

Table S4: Weighted Distribution of Sample Characteristics by Colorectal Cancer Screening Status Among U.S. Males Aged 50–75

	Overall Counts (N = 246,010)			Screened for Colorectal Cancer (Male) Yes (N = 182,834)		Screened for Colorectal Cancer (Male) No (N = 63,176)		
	Unweighted Counts	Weighted Median	Weighted IQR	Weighted Median	Weighted IQR	Weighted Median	Weighted IQR	P Value*
**Age at survey**	246,010	59.47	53.91 - 65.23	60.87	55.50 - 66.49	55.62	51.58 - 61.60	<.0001

	Overall Counts (N = 246,010)			Screened for Colorectal Cancer (Male) Yes (N = 182,834)		Screened for Colorectal Cancer (Male) No (N = 63,176)		P Value**
	Unweighted Counts	Weighted Percent	95% CI for Percent	Weighted Percent	95% CI for Percent	Weighted Percent	95% CI for Percent	
**Currently Have Asthma**								
Yes	16,343	6.44	6.23 - 6.64	6.87	6.62 - 7.11	5.4	5.03 - 5.78	<.0001
No	229,667	93.56	93.36 - 93.77	93.13	92.89 - 93.38	94.6	94.22 - 94.97	
**Race**								
White, Non-Hispanic	205,810	76.24	75.81 - 76.68	78	77.51 - 78.49	72.02	71.10 - 72.94	<.0001
Black, Non-Hispanic	18,748	12.03	11.72 - 12.35	11.89	11.52 - 12.25	12.39	11.76 - 13.02	
Other Races	21,452	11.72	11.35 - 12.10	10.12	9.71 - 10.52	15.58	14.75 - 16.42	
**Education**								
Less than high school graduate	15,283	12.19	11.83 - 12.56	9.27	8.89 - 9.64	19.25	18.41 - 20.09	<.0001
High school graduate or GED	66,935	27.7	27.32 - 28.08	26.05	25.62 - 26.48	31.66	30.87 - 32.45	
Some college or technical school	64,979	29.86	29.44 - 30.28	30.8	30.31 - 31.29	27.59	26.76 - 28.41	
College graduate or more	98,813	30.25	29.88 - 30.62	33.88	33.43 - 34.32	21.5	20.86 - 22.14	
**Employment Status**								
Employed for wages or self-employed	134,394	57.69	57.25 - 58.12	54.39	53.89 - 54.89	65.63	64.79 - 66.47	<.0001
Homemaker, student, or retired	81,688	28.32	27.93 - 28.71	33.08	32.61 - 33.56	16.85	16.20 - 17.49	
Out of work	9,714	4.8	4.59 - 5.01	3.77	3.55 - 3.98	7.28	6.79 - 7.78	
Unable to work	20,214	9.19	8.93 - 9.45	8.76	8.46 - 9.05	10.24	9.71 - 10.76	
**Income**								
<$15,000	19,074	8.41	8.14 - 8.67	6.76	6.47 - 7.05	12.38	11.80 - 12.95	<.0001
$15,000 to less than $25,00	29,670	12.31	12.01 - 12.60	10.69	10.38 - 10.99	16.21	15.54 - 16.89	
$25,000 to less than $35,00	22,597	9.2	8.92 - 9.48	8.26	7.96 - 8.55	11.45	10.82 - 12.09	
$35,000 to less than $50,00	31,699	12.11	11.82 - 12.40	11.69	11.37 - 12.00	13.12	12.50 - 13.75	
$50,000 or more	142,970	57.98	57.54 - 58.42	62.6	62.11 - 63.10	46.83	45.94 - 47.72	
**Marital Status**								
Married or member of an unmarried couple	161,161	68.41	68.01 - 68.81	71.83	71.39 - 72.27	60.18	59.34 - 61.03	<.0001
Never married	25,222	9.56	9.30 - 9.82	8.04	7.76 - 8.32	13.22	12.66 - 13.79	
Separated, divorced, or widowed	59,627	22.03	21.68 - 22.38	20.13	19.75 - 20.52	26.59	25.86 - 27.33	
**Health Insurance Coverage**								
Yes	231,719	92.47	92.19 - 92.76	96.15	95.93 - 96.38	83.62	82.86 - 84.37	<.0001
No	14,291	7.53	7.24 - 7.81	3.85	3.62 - 4.07	16.38	15.63 - 17.14	
**Smoking Status**								
Current smoker	38,275	16.59	16.26 - 16.92	13.25	12.92 - 13.59	24.62	23.83 - 25.40	<.0001
Former smoker	84,384	33.48	33.06 - 33.89	35.76	35.27 - 36.24	27.99	27.21 - 28.78	
Never smoker	123,351	49.93	49.49 - 50.38	50.99	50.48 - 51.40	47.39	46.50 - 48.29	
**Physical Activity for Leisure in Past 30 Days**								
Yes	188,758	75.55	75.16 - 75.94	77.72	77.29 - 78.15	70.31	69.48 - 71.14	<.0001
No	57,252	24.45	24.06 - 24.84	22.28	21.85 - 22.71	29.69	28.86 - 30.52	
**Heavy Alcohol Consumption (Male > 14 drinks/week; Female > 7 drinks/week)**								
Yes	16,645	6.51	6.31 - 6.71	5.9	5.68 - 6.13	7.97	7.56 - 8.39	<.0001
No	229,365	93.49	93.29 - 93.69	94.1	93.87 - 94.32	92.03	91.61 - 92.44	
**Depression**								
Yes	33,941	13.25	12.96 - 13.53	14.04	13.70 - 14.37	11.34	10.80 - 11.88	<.0001
No	212,069	86.75	86.47 - 87.04	85.96	85.63 - 86.30	88.66	88.12 - 89.20	
**Obesity**								
Obese	89,381	36.61	36.18 - 37.03	37.45	36.96 - 37.94	34.58	33.72 - 35.45	<.0001
Not obese	156,629	63.39	62.97 - 63.82	62.55	62.06 - 63.04	65.42	64.55 - 66.28	

Footnotes:

* P-value calculated using the Wald test.

** P-value calculated using the Rao–Scott chi-square test.
