# Supplementary material for: Prevalence of Adult Asthma and History of Screening for Cancer Among US Adults: Results from 2016, 2018, 2020, and 2022 National Level Cross-Sectional Study
Source: Int J Environ Res Public Health. 2025 Dec 23;23(1):23. doi: 10.3390/ijerph23010023 (PMC12840605; doi:10.3390/ijerph23010023)
Supplement: Supplementary file 1 [file ijerph-23-00023-s001.zip › Table S5.pdf]

Table S5: Weighted Distribution of Sample Characteristics by Colorectal Cancer Screening Status Among U.S. Females Aged 50–75

	Overall Counts (N = 272,334)			Screened for Colorectal Cancer (Female) Yes (N = 212,729)		No (N = 59,605)		
	Unweighted Counts	Weighted Median	Weighted IQR	Weighted Median	Weighted IQR	Weighted Median	Weighted IQR	P Value*
**Age at survey**	272,334	59.79	54.18 - 65.88	61.11	55.58 - 66.90	55.49	51.51 - 61.79	<.0001

	Overall Counts (N = 272,334)			Screened for Colorectal Cancer (Female) Yes (N = 212,729)		No (N = 59,605)		P Value**
	Unweighted Counts	Weighted Percent	95% CI for Percent	Weighted Percent	95% CI for Percent	Weighted Percent	95% CI for Percent	
**Currently Have Asthma**								
Yes	33,593	12.35	12.06 - 12.64	12.87	12.54 - 13.20	10.76	10.19 - 11.34	<.0001
No	238,741	87.65	87.36 - 87.94	87.13	86.80 - 87.46	89.24	88.66 - 89.81	
**Race**								
White, Non-Hispanic	223,867	76.25	75.81 - 76.68	76.94	76.45 - 77.43	74.16	73.20 - 75.11	<.0001
Black, Non-Hispanic	27,610	13.64	13.33 - 13.95	13.96	13.59 - 14.32	12.67	12.05 - 13.28	
Other Races	20,857	10.11	9.74 - 10.49	9.1	8.69 - 9.51	13.18	12.30 - 14.05	
**Education**								
Less than high school graduate	14,396	10.28	9.94 - 10.62	8.75	8.40 - 9.10	14.9	14.05 - 15.75	<.0001
High school graduate or GED	69,167	26.45	26.07 - 26.83	25.6	25.17 - 26.02	29.03	28.20 - 29.87	
Some college or technical school	80,027	33.03	32.61 - 33.46	33.74	33.25 - 34.22	30.91	30.01 - 31.80	
College graduate or more	108,744	30.24	29.86 - 30.62	31.92	31.48 - 32.35	25.16	24.39 - 25.94	
**Employment Status**								
Employed for wages or self-employed	123,042	47.58	47.13 - 48.02	44.88	44.39 - 45.38	55.73	54.78 - 56.68	<.0001
Homemaker, student, or retired	112,639	37.12	36.69 - 37.54	40.57	40.08 - 41.05	26.67	25.83 - 27.51	
Out of work	9,822	4.49	4.29 - 4.70	3.85	3.63 - 4.07	6.44	5.95 - 6.92	
Unable to work	26,831	10.82	10.55 - 11.08	10.7	10.40 - 11.00	11.17	10.62 - 11.72	
**Income**								
<$15,000	26,993	10.56	10.28 - 10.84	9.36	9.05 - 9.66	14.2	13.52 - 14.88	<.0001
$15,000 to less than $25,00	41,386	14.94	14.63 - 15.25	13.83	13.49 - 14.17	18.31	17.57 - 19.04	
$25,000 to less than $35,00	29,210	10.39	10.12 - 10.65	10.27	9.97 - 10.56	10.74	10.18 - 11.31	
$35,000 to less than $50,00	38,123	12.79	12.49 - 13.08	12.92	12.60 - 13.24	12.38	11.70 - 13.05	
$50,000 or more	136,622	51.33	50.89 - 51.77	53.63	53.13 - 54.12	44.38	43.42 - 45.33	
**Marital Status**								
Married or member of an unmarried couple	153,260	59.57	59.13 - 60.00	60.66	60.17 - 61.14	56.27	55.32 - 57.23	<.0001
Never married	22,383	7.94	7.69 - 8.20	7.19	6.92 - 7.46	10.23	9.62 - 10.83	
Separated, divorced, or widowed	96,691	32.49	32.08 - 32.90	32.15	31.69 - 32.61	33.5	32.61 - 34.40	
**Health Insurance Coverage**								
Yes	260,544	94.39	94.16 - 94.61	96.64	96.44 - 96.83	87.57	86.90 - 88.24	<.0001
No	11,790	5.61	5.39 - 5.84	3.36	3.17 - 3.56	12.43	11.76 - 13.10	
**Smoking Status**								
Current smoker	39,448	14.8	14.50 - 15.09	12.67	12.36 - 12.98	21.22	20.49 - 21.95	<.0001
Former smoker	75,556	26.66	26.28 - 27.04	28.27	27.83 - 28.71	21.81	21.05 - 22.56	
Never smoker	157,330	58.54	58.11 - 58.97	59.06	58.58 - 59.54	56.97	56.04 - 57.90	
**Physical Activity for Leisure in Past 30 Days**								
Yes	200,180	72.22	71.81 - 72.62	73.31	72.86 - 73.75	68.92	68.01 - 69.83	<.0001
No	72,154	27.78	27.38 - 28.19	26.69	26.25 - 27.14	31.08	30.17 - 31.99	
**Heavy Alcohol Consumption (Male > 14 drinks/week; Female > 7 drinks/week)**								
Yes	17,498	6.27	6.07 - 6.48	6.27	6.04 - 6.51	6.28	5.84 - 6.71	0.992
No	254,836	93.73	93.52 - 93.93	93.3	93.49 - 93.96	93.72	93.29 - 94.16	
**Depression**								
Yes	65,869	23.66	23.30 - 24.02	24.88	24.46 - 25.30	19.96	19.27 - 20.66	<.0001
No	206,465	76.34	75.98 - 76.70	75.12	74.70 - 75.54	80.04	79.34 - 80.73	
**Obesity**								
Obese	97,341	36.05	35.62 - 36.47	36.53	36.05 - 37.00	34.58	33.67 - 35.50	0.0002
Not obese	174,993	63.95	63.53 - 64.38	63.47	63.00 - 63.95	65.42	64.50 - 66.33	

Footnotes:

* P-value calculated using the Wald test.

** P-value calculated using the Rao–Scott chi-square test.
